# Rare Opportunities for Insights Into Serotonergic Contributions to Brain and Bowel Disorders: Studies of the SERT Ala56 Mouse

**DOI:** 10.3389/fncel.2021.677563

**Published:** 2021-06-03

**Authors:** Samantha E. Stilley, Randy D. Blakely

**Affiliations:** ^1^Department of Biomedical Science, Charles E. Schmidt College of Medicine, Florida Atlantic University, Boca Raton, FL, United States; ^2^Brain Institute, Florida Atlantic University, Jupiter, FL, United States

**Keywords:** serotonin, autism, SERT, SERT Ala56 mouse, p38α MAPK, immune, IL-1β

## Abstract

Altered structure, expression, and regulation of the presynaptic serotonin (5-HT) transporter (SERT) have been associated with multiple neurobehavioral disorders, including mood disorders, obsessive-compulsive disorder (OCD), and autism spectrum disorder (ASD). Opportunities to investigate mechanistic links supporting these associations were spurred with the identification of multiple, rare human SERT coding variants in a study that established a male-specific linkage of ASD to a linkage marker on chromosome 17 which encompassed the location of the SERT gene (*SLC6A4*). We have explored the most common of these variants, SERT Ala56, *in vitro* and *in vivo*. Results support a tonic elevation of 5-HT transport activity in transfected cells and human lymphoblasts by the variant *in vitro* that leads to an increased 5-HT clearance rate *in vivo* when studied in the SERT Ala56 mouse model, along with altered sensitivity to SERT regulatory signaling pathways. Importantly, hyperserotonemia, or an elevated whole blood 5-HT, level, was found in SERT Ala56 mice, reproducing a well-replicated trait observed in a significant fraction of ASD subjects. Additionally, we found multiple biochemical, physiological, and behavioral alterations in the SERT Ala56 mice that can be analogized to those observed in ASD and its medical comorbidities. The similarity of the functional impact of the SERT Ala56 variant to the consequences of p38α MAPK activation, ascribed to the induction of a biased conformation of the transporter toward an outward-facing conformation, has resulted in successful efforts to restore normal behavioral and bowel function *via* pharmacological and genetic p38α MAPK targeting. Moreover, the ability of the inflammatory cytokine IL-1β to enhance SERT activity *via* a p38α MAPK-dependent pathway suggests that the SERT Ala56 conformation mimics that of a chronic inflammatory state, supporting findings in ASD of elevated inflammatory cytokine levels. In this report, we review studies of the SERT Ala56 variant, discussing opportunities for continued insight into how chronically altered synaptic 5-HT homeostasis can drive reversible, functional perturbations in 5-HT sensitive pathways in the brain and periphery, and how targeting the SERT regulome, particularly through activating pathways such as those involving IL-1β/p38α MAPK, may be of benefit for neurobehavioral disorders, including ASD.

## Introduction: Serotonergic Connections to ASD

Infantile autism was first described by Leo Kanner, who reported the behavioral features of 11 children who were unable to engage with others socially or to understand the intent of those around them, preferring aloneness as well as sameness (Kanner, 1943). Kanner also noticed that these traits seemed to be ones the children were born with, rather than developing over time. Today, what Kanner referred to as autism is recognized to exemplify significant phenotypic heterogeneity, in keeping with a formal diagnosis of autism spectrum disorder (ASD). Given its heterogeneity of presentation and traits that can be seen in other disorders, ASD can only be formally diagnosed through rigorous behavioral assessments, with two domains of deficits recognized by the fifth edition of the Diagnostic and Statistical Manual of Mental Disorders (DSM-5). These domains are described as exhibiting: (1) persistent deficits in social communication and social interaction across multiple contexts; and (2) restricted, repetitive patterns of behavior, interests, or activities. For diagnosis, these symptoms must be present in the early developmental period and lead to clinically significant impairment in social, occupational, or other important areas of current functioning, with features not better explained by intellectual disability or global developmental delay (American Psychiatric Association, 2000). Symptoms often appear before 3 years of age and are lifelong, leading to a very high socioeconomic burden for parents and other caregivers. ASD is a widely prevalent disease affecting 1 in 54 children and is more common in boys (Merikangas and Alsamy, 2020). Because the definition and diagnostic labeling of ASD has changed since its original description, we will use the term ASD in this review, doing so with the clear recognition that this term was not used in the earlier studies we note.

With little understanding of the basis of ASD at the time of discovery, a potentially exciting lead appeared with the finding that some patients presented with high levels of serotonin (5-HT) in the blood, or hyperserotonemia (Schain and Freedman, 1961). Blood 5-HT derives from enterochromaffin cells that release the amine for local actions, but which is also released into the enteric circulation, where it first encounters platelets that express high levels of the high-affinity, antidepressant-sensitive 5-HT transporter (SERT; Erspamer and Asero, 1952; Anderson et al., 1987). Because free 5-HT in the blood is rapidly metabolized and cleared from the blood, and platelet number has not been found to be elevated, the hyperserotonemia of ASD is believed to represent elevated platelet stores of 5-HT (Veenstra-VanderWeele et al., 2009). Hyperserotonemia can be identified in approximately 30% of ASD patients and to date, remains the most reproducible biomarker associated with ASD (Pagan et al., 2021). However, the presence of the trait in some individuals that do not meet criteria for ASD (Takahashi et al., 1976a, b; Hanna et al., 1991) and its lack of representation in a majority of ASD subjects precludes hyperserotonemia from being diagnostically determinative. It is reported that hyperserotonemia of ASD is more common in boys than in girls (Shuffrey et al., 2017), as is ASD, suggesting a connection to mechanisms that support pathophysiology, though exact mechanisms remain ill-defined. Further evidence that altered 5-HT homeostasis or sensitivity plays a role in ASD derives from studies demonstrating that 5-HT selective reuptake inhibitors mitigate symptoms of receptiveness and aggressiveness in ASD subjects (Mehlinger et al., 1990; Todd, 1991; Cook et al., 1992; Cook and Leventhal, 1996), though the core features of ASD are not overcome (King et al., 2009). Notably, when 5-HT levels are depleted using tryptophan depletion, ASD symptoms are worsened (McDougle et al., 1993, 1996).

Familial relationships of hyperserotonemia have been reported (Leventhal et al., 1990) though whether this indicates common heritability or environmental influence is not clear. However, findings report that affected siblings with ASD have higher levels of serotonin than an affected ASD subject without another sibling that is affected, supporting heritability (Piven et al., 1991). Indeed, further studies provided evidence that blood 5-HT level is a highly heritable trait (Leventhal et al., 1990; Weiss et al., 2005; Muller et al., 2016), indicating that the changes found in hyperserotonemic ASD subjects may have identifiable genetic determinants. Because platelets do not synthesize 5-HT but acquire it from the bloodstream *via* SERT, and because a single gene expresses SERT found in both the brain and platelets (Lesch et al., 1993; Ramamoorthy et al., 1993) the transporter has been a major focus for models seeking to connect hyperserotonemia to ASD (Rotman et al., 1980; Piven et al., 1991; Cook and Leventhal, 1996).

Evidence of 5-HT (Wallace and Lauder, 1983) and SERT (Altamura et al., 2007; Chen et al., 2015) actions during brain development has bolstered considerations of the transporter as relevant for neurodevelopmental disorders such as ASD. During development, SERT is expressed in glutamatergic thalamocortical axons and influences sensory map architecture. SERT knock-out in these neurons but not in serotonergic neurons causes lasting changes in sensory map architecture (Chen et al., 2015). Changes in the thalamocortical projections could contribute to sensory hypersensitivity that is seen in individuals with ASD and other neurodevelopmental disorders (Chen et al., 2015). Significant effects of 5-HT manipulations on fetal neurogenesis have also been noted (Lauder and Krebs, 1978). 5-HT synthesis, quantity, and receptor binding sites are highest during development and are then reduced around puberty (Chugani et al., 1999). Interestingly, the temporal profile of developmental 5-HT synthesis appears altered in ASD patients, consistent with a developmental disturbance in 5-HT homeostasis in these children (Chugani et al., 1999). With the cloning of rodent SERTs (Blakely et al., 1991; Hoffman et al., 1991; Chang et al., 1996) and the availability of gene expression and antibody probes to localize sites of SERT mRNA and protein expression in rodents, we (Schroeter and Blakely, 1996) and others (Hansson et al., 1998; Lebrand et al., 1998, 2006) found significant expression of the transporter occurring in mouse and brain serotonergic neurons in the midgestational embryo, with transient expression in non-serotonergic neurons. The non-serotonergic sites include glutamategeric thalamocortical neurons (Hansson et al., 1998) and prefrontal cortex (PFC) neurons (Soiza-Reilly et al., 2019) where expression continues into the early postnatal period. Moreover, Chen and colleagues have shown that early developmental SERT expression in glutamatergic thalamocortical neurons is critical for proper development of somatosensory cortex (Chen et al., 2015) whereas Soiza-Reilly and coworkers demonstrated a requirement for developmental SERT expression by PFC neurons in the establishment of normal excitability of dorsal raphe neurons (5-HT and non 5-HT) and behavioral responses to stress. Lastly, work by Bonnin and colleagues demonstrated that significant levels of 5-HT exist in the developing mouse forebrain prior to the region’s enervation by serotonergic axons, derived from the placenta (Bonnin et al., 2011) speaking to even earlier roles for 5-HT in brain development. Together, these studies argue strongly that a focus on adult-onset mood disorders in considering a role for SERT in neurobehavioral disorders is myopic, with a more developmental disorder orientation reasonable in the light of the aforementioned links of 5-HT and SERT to ASD.

## SERT: from Gene to Genetic Studies of ASD

As alluded to above, the vast majority of studies considering a role for SERT in pathophysiological states concern a role in mood and anxiety disorders and 5-HT uptake attenuation by 5-HT-selective reuptake inhibitors (SSRIs; Montgomery, 1995; Owens and Nemeroff, 1998). This body of work encouraged our efforts to clone human SERT cDNA (Ramamoorthy et al., 1993) and implement chromosomal *in situ* hybridization to provide evidence for a single locus for the cognate gene (*SLC6A4*) at 17q11.2, a finding subsequently validated by the Human Genome Project. The predicted sequence of human SERT and its evolutionary variants suggests a transporter protein structure comprised of 12 transmembrane domains (TMs) with intracellular NH2 and COOH termini. This prediction has been amply verified through biochemical studies and most importantly through the generation of high-resolution X-ray crystal structures of human SERT in different conformations bound to 5-HT and antagonists (Coleman et al., 2016, 2019; Coleman and Gouaux, 2018). The sequence encoding human SERT protein is actually a small fraction of the size of the *SLC6A4* gene (~2 kb vs. ~40 kb), with significant opportunities for noncoding regulation at the genomic and mRNA level (Bradley and Blakely, 1997; Murphy and Moya, 2011). Two common, non-coding polymorphisms in the *SLC6A4* gene have been the subject of many studies seeking to link SERT expression to genetic variation and neurobehavioral disorders, an intron 2 variable nucleotide tandem repeat (STin2; Lesch et al., 1994) and a promoter region insertion-deletion polymorphism (5-HTTLPR; Lesch et al., 1996) In relation to ASD, previous studies have suggested that no significant relationship between these polymorphisms and ASD can be established and it is unlikely that they play a significant role in the susceptibility to ASD (Betancur et al., 2002; Huang and Santangelo, 2008). However, other studies have reported that these polymorphisms increase the risk for hyperserotonemia in autistic patients, which and therefore may contribute to behavioral manifestations in a subset of subjects (Coutinho et al., 2004; Jaiswal et al., 2015).

## Rare Opportunities to Assess A Serotonergic Contribution to ASD: SERT Coding Variation

Mechanistic insights concerning the impact of common (or rare) non-coding genetic variation *in vivo* can be particularly challenging given a lack of evolutionary conservation that is often seen when moving from human to model systems such as mouse or rat and difficulties in developing *in vitro* models, particularly considering the large size of many genes, including most of the transporters found in the SLC6 transporter family. In contrast, the identification of disease-associated coding variation presents a particularly important opportunity to identify functional impact as the sites within a protein where such variation is found are often highly conserved and can be both studied *in vitro* using mutant cDNAs and *in vivo* through simple codon substitutions. Initial searches for common, disease-associated coding variation in *SLC6A4* failed to indicate common coding variation that could be linked to neurobehavioral disorders including unipolar depression, bipolar disorder (Lesch et al., 1995) and obsessive-compulsive disorder (OCD; Altemus et al., 1996) with a single instance of a coding variant (Leu255Met) found among 67 subjects with major depression (Di Bella et al., 1996) or an additional 74 with OCD, and, due to its rarity and location distant from presumed functional domains, was not further characterized. Consistent with these findings, a much larger analysis of GenBank deposited cDNA sequences revealed nine *SLC6A4*-associated coding variants in 450 subjects, although each was found in single individuals, with the exception of an N-terminal-localized Gly56Ala substitution found in four subjects (frequency = 0.009; Glatt et al., 2001). In general, consistent findings of an exceedingly low frequency of coding variation in the *SLC6A4* gene encouraged the focus on association studies that could be pursued with much more common promoter and intronic variation.

Undeterred by the rarity of SERT coding variation, or perhaps prescient that such variation, if disease-associated, might contribute to disorders presenting with a more heterogenous phenotype, Murphy’s group pursued sequencing of families presenting with multiple members who exhibit a spectrum of neurobehavioral disorders including OCD, AS (Asperger’s syndrome), anorexia and substance use disorders (Ozaki et al., 2003). Multiple affected individuals from two unrelated families were found to express a SERT Ile425Val coding variant, modifying a highly conserved position in the eighth transmembrane domain, and resulting in elevated 5-HT transport activity in transfected COS-7 cells (Kilic et al., 2003). Wendland and colleagues (Wendland et al., 2008) pursued a much larger case-control study of functional variants in OCD and found none to carry the 425Val variant, indicating that the disorder, like other neuropsychiatric disorders, is etiologically heterogeneous. Similar conclusions were reached from a large multicenter family genotyping study, where three individuals out of 1241 individuals were found to carry the Val425 variant, two with OCD diagnoses, though none transmitted the variant to their six affected children (Voyiaziakis et al., 2011), suggesting that the variant, though functional *in vitro*, may not act deterministically *in vivo*, at least in subjects with a highly defined OCD diagnosis. Regardless, the findings by Ozaki et al. of SERT coding variation noted above raised the possibility that consideration of phenotypes within broad spectrum disorders like ASD, or across diagnostically distinct disorders, particularly those with OCD-like traits, might be more likely to present with functionally penetrant transporter mutations.

Sutcliffe’s group (McCauley et al., 2004) pursued linkage studies in 137 families bearing ASD offspring, identifying a significant male-specific signal at 17q11.2, an area containing the *SLC6A4* gene. These findings were of interest as *SLC6A4* had previously been identified with a male-specific linkage to whole blood 5-HT levels at 17q (Weiss et al., 2005). The *ITGB3* gene, encoding the β3 subunit of the fibrinogen receptor (integrin αIIbβ3) that is highly expressed on platelets, is localized to 17q21.3, and was also identified in the Weiss et al study as a quantitative trait locus (QTL) for whole blood 5-HT. Interestingly, Carneiro and colleagues demonstrated that SERT and integrin αIIbβ3 can be isolated in a physical complex in platelets (Carneiro et al., 2008) and that fibrinogen produces SERT activation *via* a p38 MAPK pathway. Moreover when the *Itgb3* gene is manipulated in mice, 5-HT synapse density is altered (Dohn et al., 2017), as is synaptosomal 5-HT uptake (Mazalouskas et al., 2015), and ASD-like behaviors emerge (Dohn et al., 2017).

**Figure 1:**
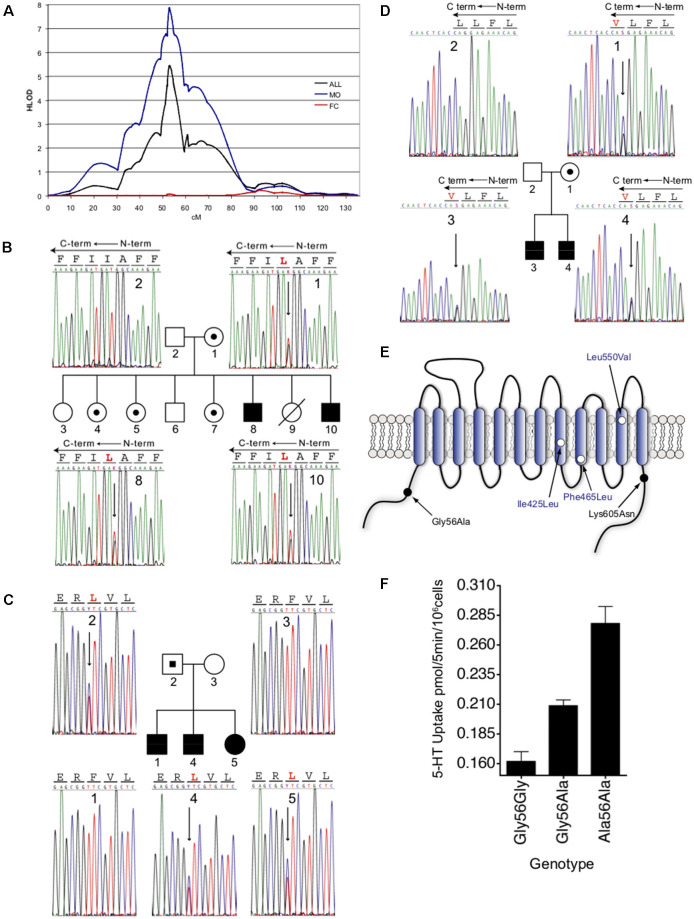
Male-biased linkage of autism and novel coding variants at the 17q11.2 *SLC6A4* locus. **(A)**. Male-biased linkage of autism to 17q11.2. Multipoint linkage analysis on chromosome 17 is shown for the overall 341-family data set (*black line*), 202 MO families (*blue line*), or the remaining 138 FC families (*red line*). HLOD scores were calculated under a recessive model and were plotted as a function of marker position in centimorgans (cM) along chromosome 17. **(B–D)** Sequence detection of novel nonsynonymous *SLC6A4* variants in families with autism. Sequence-based detection is shown for each of the three novel coding variants, with corresponding pedigrees. **(B)** Ile425Leu. **(C)** Phe465Leu. **(D)** Leu550Val. Blackened circles or squares reflect individuals with an autism diagnosis, unblackened circles or squares reflect individuals without autism, and allele carriers without autism are indicated by small blackened circles or squares within the larger pedigree symbol. Electropherogram data is shown in either sense **(B)** or antisense **(A,C)** orientations, with corresponding coding sequence. Antisense sequences **(A,C)** indicate the reversed orientation of amino acid codons, represented by lines across each three-base sequence. Variant amino acids are shown in red, and corresponding heterozygous sequence changes are indicated by an arrow. Individual numbers in the respective pedigrees correspond to numbers within each of the sequence frames. **(E)** Schematic representation of the 5-HT transporter. Amino acid substitutions are indicated by location within transmembrane or cytoplasmic domains. **(F)** Dosage-dependent elevated 5-HT transporter activity of Ala56-encoded SERT in native lymphoblastoid cells. Lymphocytes genotype-matched at 5-HTTLPR (L/L) and the intron 2 VNTR (10/10) and bearing Gly/Gly, Gly/Ala, or Ala/Ala encoding genotypes at residue 56 were assayed for [^3^H]5-HT transport activity. Three independent experiments were performed in triplicate for each line, and the combined basal uptake data were plotted. Figure from Sutcliffe et al. (2005).

When again referring to the linkage study of McCauley et al. (2004) an increased linkage was seen in the 70 families of this collection possessing probands that exhibited rigid-compulsive traits (RCTs), with an absence of linkage evident in families lacking probands with RCTs, consistent with a model of different genes contributing to the varying traits of ASD (e.g., development and use of language, social behavior dysfunction, sensory aversion, RCTs). Only nominal association with these traits was seen for the 5-HTTLPR (short allele), suggesting that if mutations in *SLC6A4* contributed to the linkage, a model of over-transmission of heterogeneous, functional alleles was more plausible. In an expanded set of 341 families (Sutcliffe et al., 2005), the group reinforced the 17q11.2 linkage finding and its sex-dependence, with linkage at this region eliminated when families with at least one affected female (FC) were analyzed separately and strengthened when considering families with only male probands (MO) (Figure 1A). In evaluating families most contributing to the linkage, four SERT coding variants (familial transmission of three variants noted in Figures 1B–D), and 15 other novel noncoding variants, were identified, with a fifth coding variant identified in an expanded set of families (Figure 1E). Given the low frequency of coding variants exhibited by the *SLC6A4* gene, the abundance of such variants in the Sutcliffe et al. study deserved further scrutiny. The most common of the coding variants identified was the Gly56Ala substitution that had been previously identified (Glatt et al., 2001). Although relatively few in number, the Ala56 encoding allele was found to be associated with ASD in the assembled families, was more frequent in affected males, and was over-transmitted to offspring with ASD. Subjects expressing this variant were enriched for traits of altered interpretation of social intent, RCTs, and sensory aversion when compared to the full ASD group (Sutcliffe et al., 2005). It should be noted that Sakurai and colleagues, using a large–scale screening approach with a different family structure, also identified G56A in 4/350 cases (Sakurai et al., 2008), a difference in frequency not different from controls. The other substitutions identified in the Sutcliffe et al.’s (2005) study included Ile425Leu, Phe465Leu, Leu550Val, and Lys605Asn, all occurring at highly conserved positions. The identification of the Leu425 coding variant is particularly striking given the prior (Ozaki et al., 2003) and subsequent findings of the Val425 variant (Moya et al., 2013) in subjects with OCD traits and Tourette’s Disorder traits.

Given the overall rarity of the SERT coding variants identified in ASD or phenotypically somewhat related disorders (ASD and OCD both presenting with RCTs) and their conditional penetrance *in vivo*, the degree to which they speak to serotonergic dysfunction in these disorders rests significantly on their ability to alter SERT function, and ultimately, 5-HT signaling and behavior. Sutcliffe and colleagues (Sutcliffe et al., 2005) reported that the SERT Ala56 variant conferred elevated 5-HT uptake activity to lymphoblasts derived from ASD subjects in the linkage study, with homozygous carriers exhibiting the highest activity (Figure 1F). Similar elevations of lymphoblast 5-HT uptake could be shown in studies with the other coding variants noted, with no significant differences found in glutamate uptake (Prasad et al., 2009). Heterologous expression studies (Prasad et al., 2005, 2009; Sutcliffe et al., 2005) revealed that Ala56, as well as the Leu or Val425, Leu465, and Val550 variants, demonstrate increased 5-HT uptake compared to wildtype SERT (Ye and Blakely, 2011). These findings indicate that the variants themselves confer uptake elevations, rather than other aspects of the lymphoblast environment. All variants, except Ala56, demonstrated elevated surface SERT expression (Prasad et al., 2005, 2009). SERT Ala56 appears to achieve elevated 5-HT uptake stimulation through catalytic activation, derived from a decreased 5-HT K_M_. *In toto*, these findings led to the hypothesis that reduced synaptic 5-HT availability during development may impact risk for the development of ASD traits (Ye and Blakely, 2011). Subsequent studies have confirmed this and shown that a maternal SERT Ala56 genotype affects offspring forebrain 5-HT levels and broadens the thalamocortical axon projections (Muller et al., 2017). Thus, the suggestion that SERT coding variation impacts brain development, given the evidence of genetic linkage and functional consequences to 5-HT uptake, dovetails with the identified embryonic expression of SERT, and that 5-HT serves as an axonal guidance molecule in the developing brain (Haydon et al., 1984; Bonnin et al., 2007).

## Elevation of SERT Activity by Activation of PKG and P38α MAPK-Linked Pathways

**Figure 2:**
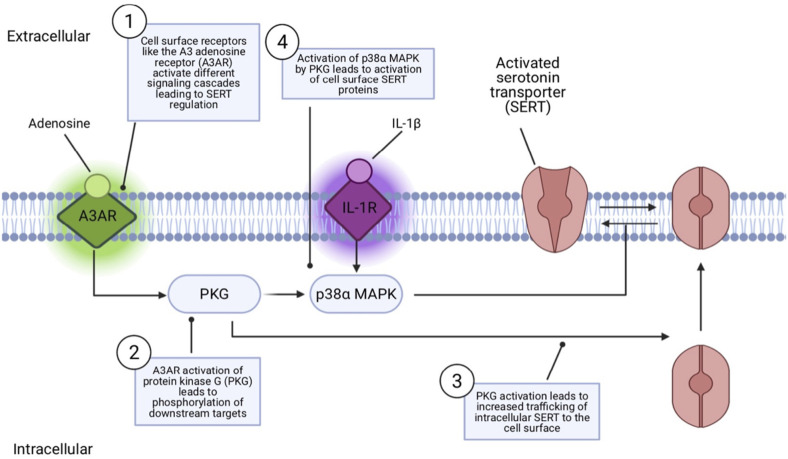
Model of receptor-mediated SERT regulation. Cell surface receptors like the adenosine receptor (A3AR) can regulate SERT *via* a protein kinase G (PKG) pathway that can both mobilize intracellular SERT proteins to move to the cell surface and, *via* a second, p38α MAPK-linked pathway, activate surface transporters to rapidly increase 5-HT transport rates. IL-1R stimulation by the inflammatory cytokine Il-1β can lead to SERT activation *via* PKG-independent p38α MAPK stimulation, without impacting SERT surface trafficking.

Findings that SERT coding variation identified in ASD subjects impacts SERT activity raise questions as to how SERT naturally changes functional state or is regulated in its routing to and from the cell surface. Significant research has revealed that SERT activity and surface activity can be rapidly and independently regulated by multiple G protein-coupled receptors and protein kinases (Ramamoorthy et al., 2011; Bermingham and Blakely, 2016). These include protein kinase C (PKC; Qian et al., 1997; Jayanthi et al., 2005), protein kinase G (PKG; Zhu et al., 2004; Ramamoorthy et al., 2007; Chang et al., 2012), and p38 MAPK (Zhu et al., 2004, 2006; Prasad et al., 2005). Activation of PKC results in an internalization of SERT which results in blunted serotonin uptake (Qian et al., 1997). PKC activators rapidly, and in a concentration-dependent manner, elevate basal hSERT phosphorylation. Protein phosphatases are also regulators of SERT, as initially shown with studies of PP1/2A inhibitors that downregulate 5HT transport and elevate phosphorylation (Ramamoorthy et al., 1998). These studies led to studies demonstrating a complex formed between SERT and PP2A (Bauman et al., 2000). PKG have been shown to increase cell surface SERT protein, increase phosphorylation, and increase SERT uptake (Zhu et al., 2004). Importantly, PKG and p38 MAPK regulatory pathways have been found to lie downstream of multiple cell surface receptors. Specifically, adenosine A3 receptors (A3ARs) have been found to induce a rapid increase in SERT activity, mediated by PKG-dependent surface trafficking as well as by PKG- and p38 MAPK-dependent catalytic activation in RBL-2H3 cells (Zhu et al., 2004, 2007) and serotonergic nerve terminals (Zhu et al., 2007). The pro-inflammatory cytokine IL-1β has been found to rapidly activate cell surface SERT proteins catalytically *in vitro* and *in vivo* *via* IL-1R1 receptors (Zhu et al., 2006) providing a link between innate immune system activation and changes in serotonergic signaling, just one of the paths of cross-talk between the immune system and 5-HT signaling (Baganz and Blakely, 2013). This IL-1R1 dependent activation of SERT involves an IL-1R1 to p38α MAPK signaling pathway and can be induced rapidly (1 h) in the brain by peripheral innate immune system activation by i.p. lipopolysaccharide (LPS; Figure 2; Zhu et al., 2010). Conditional deletion of p38α MAPK in serotonin neurons results in a loss of the ability of peripheral LPS to stimulate brain SERT as well as to trigger SSRI-sensitive despair-like behavior as well as anxiety-like traits (Baganz et al., 2015).

## Altered Sensitivity of SERT Coding Variants to Pkg and P38α MAPK Signaling

**Figure 3:**
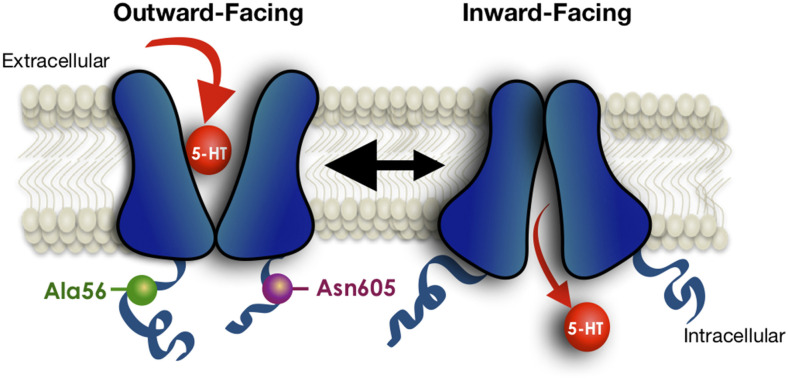
Schematic indicating conformational bias of SERT Ala56 and Asn605 relative to WT SERT. Limited proteolysis, fluorescence resonance energy transfer (FRET), and cysteine-accessibility studies indicate that the Ala56 and Asn605 coding variants impose conformation that leading to a more outward-facing conformation of the transporter, facilitating access of 5-HT to the substrate-binding site under resting conditions. Modified from Quinlan et al. (2019).

To gain a broader understanding of the functional impact of human SERT coding variation, Prasad and colleagues (Prasad et al., 2005) sought evidence of 5-HT transport changes associated with the known variants at the time. Several variants were found to cause an elevation in 5-HT uptake in transfected HeLa cells, including SERT Ala56 as noted above, and one substitution, Pro339Leu, was found to confer reduced transport uptake. The latter variant to our knowledge remains unassociated with a clinical phenotype. Given the evidence that a majority of SERT coding variants identified in ASD subjects demonstrate elevated 5-HT uptake in transfected cells, as well as in lymphoblasts of carriers (Prasad et al., 2009), and that SERT can be naturally stimulated *via* PKG and p38 MAPK-linked pathways, Prasad probed SERT variants to determine whether basal transport differences might reflect altered regulatory mechanisms. Multiple variants were found to display elevated SERT function in transfected HeLa cells due to either elevated surface expression (e.g., SERT Val425) or enhanced catalytic function (e.g., SERT Ala56; Prasad et al., 2005). Importantly, these variants also displayed either blunted or absent PKG- and p38 MAPK-mediated stimulation. Recently, Quinlan et al. (2019) provided evidence, using limited proteolysis approaches, cysteine accessibility studies, and fluorescence resonance energy transfer (FRET), that both the SERT Ala56 and Asn605 variants stabilize an open-outward conformation that can explain a constitutive shift to a higher-affinity state for 5-HT (Figure 3). SERT Ala56 also displays significantly reduced sensitivity to protein phosphatase 2A (PP2A) antagonists (Prasad et al., 2009) which may reflect the loss of PP2A complex subunits from SERT Ala56 described in recent proteomic studies (Quinlan et al., 2019). SERT exhibits changes in lateral surface mobility when regulated through PKG/p38MAPK pathways, though these changes do not appear to relocate it away from membrane rafts (Chang et al., 2012), suggesting a local untethering from associated proteins that allows for increased conformational flexibility and enhanced 5-HT transport, as seen with gain of function SERT mutants. When SERT Ala56 and SERT Leu425 are stably transfected from the same genomic locus in CHO cells, both variants display kinetic features consistent with catalytic activation (Prasad et al., 2009), consistent with findings of Kilic and Rudnick in the initial studies of the SERT Val425 variant (Kilic et al., 2003). We suggest that the surface elevation we observe with SERT Val425 expression in transfected Hela cells of elevated basal surface expression (Prasad et al., 2005), vs. the enhanced catalytic function observed for the same variant in transfected COS-7 cells (Kilic et al., 2003) derives from the relatively lower levels of expression achieved in the HeLa model vs. the COS-7 model, where significantly high expression is achieved, saturating membrane trafficking pathways, allowing only catalytic activation to occur. This idea is consistent with a model whereby normally enhanced SERT trafficking to the surface is followed rapidly by catalytic activation, driven by stabilization of an outward-facing conformation (Blakely et al., 2005), and where coding variants that lead to excess 5-HT uptake do so by driving the transporter to the membrane and/or driving an outward conformation without extrinsic kinase-activating stimuli.

## *In Vivo* Evidence of A Functional Impact of SERT Ala56 in Knock-In Mice

To more definitively establish a physiologically relevant, functional impact of the SERT Ala56 variant* in vivo*, Veenstra-VanderWeele and coworkers generated SERT Ala56 knock-in mice with homologous recombination approaches in embryonic stem cells (Veenstra-VanderWeele et al., 2009). Homozygous knock-in Ala56 mice demonstrate normal growth rates and fertility and display no gross sensorimotor deficits. As predicted by transfected cell studies, the SERT Ala56 mutation does not impact SERT protein levels as compared to wildtype littermates born from heterozygous parents (Veenstra-VanderWeele et al., 2012). Also, similar to previous findings in transfected cells, these studies revealed that SERT Ala56 phosphorylation is significantly elevated in midbrain synaptosomes under basal conditions. Moreover, activation of PKG with 8-Bromo-cGMP (8-Br-cGMP) fails to augment SERT Ala56 synaptosomal phosphorylation, whereas phosphorylation of wildtype SERT is significantly elevated. Additionally, treatment of SERT Ala56 synaptosomes with a p38 MAPK inhibitor normalizes the SERT Ala56 phosphorylation levels to that of wildtype SERT, in support of a hyperphosphorylated state of SERT Ala56 stimulated by a PKG-dependent p38 MAPK pathway. Whereas studies with SERT Ala56 synaptosomes failed to demonstrate the expected increase in basal SERT activity, studies using *in vivo* high-speed chronoamperometry revealed a significantly elevated hippocampal 5-HT clearance rate, suggesting that preparation of synaptosomes disrupts the mechanisms by which elevated SERT phosphorylation is translated into a change in catalytic function, possibly a result of cytoskeletal changes. Loose-patch recordings of dorsal raphe 5-HT neurons in midbrain slices from SERT Ala56 mice also revealed reduced basal firing rates *ex vivo*. Should these reductions occur *in vivo*, the Ala56 variant may reduce synaptic 5-HT by both decreased 5-HT release as well as enhanced 5-HT clearance. As SERT knockout mice demonstrate reductions in sensitivity of multiple 5-HT receptors (Fox et al., 2008), in keeping with excess 5-HT mediated receptor downregulation, a constitutively-imposed increased rate of 5-HT clearance is expected to increase the sensitivity of 5-HT receptors. Indeed, SERT Ala56 mice display increased sensitivity of 5-HT_1A_ and 5-HT_2A/2C_ receptors when probed *in vitro* and *in vivo* (Veenstra-VanderWeele et al., 2012). With respect to 5-HT_1A_ receptor hypersensitivity, which we demonstrated in the SERT Ala56 by monitoring hypothermia in response to the 5-HT_1A_ receptor agonist 8-OH-DPAT, we suspect that hypersensitivity of this receptor also explains the decreased firing of 5-HT neurons recorded in acute brain slices due to increased auto-inhibitory feedback. More recent studies also indicate SERT Ala56-mediated changes in 5-HT_1B_ receptor expression (O’Reilly et al., 2021). These findings provide a clear example of how a modest change in SERT structure can be amplified through changes in the multiple receptors and signaling cascades that support the actions of 5-HT *in vivo*.

## Biochemical and Behavioral Alterations in The SERT Ala56 Mouse

As noted earlier, hyperserotonemia is a well-replicated finding in a significant subset of subjects with ASD, with elevated SERT expression or activity one path to increasing platelet levels of 5-HT. SERT Ala56 mice demonstrate hyperserotonemia in comparison to their wildtype littermates (Veenstra-VanderWeele et al., 2012), the first time, to our knowledge, that this biomarker was established *in vivo* from a heritable human mutation. Most importantly, SERT Ala56 mice display behavioral changes that can be analogized to those observed in ASD. With respect to changes in language acquisition and communication ASD phenotypes, SERT Ala56 pups demonstrate reduced ultrasonic vocalization compared to wildtype siblings when separated from the mother. The mutant mice also demonstrate diminished social preference in the three-chamber test and social withdrawal in the tube test. Finally, SERT Ala56 mice display abnormal repetitive behavior, indicated by repeated cycling to the top of their cage, registered in an automated system as increased “hanging bouts” (Veenstra-VanderWeele et al., 2012). In keeping with evidence of altered sensory integration in ASD (Siemann et al., 2017, 2020), additional studies have revealed a reduced ability of SERT Ala56 mice to integrate multiple sensory cues to facilitate operant responding for reward (Siemann et al., 2017). In humans, SERT Ala56 is found in unaffected as well as ASD subjects, likely reflecting the nature of complex diseases, in which many genes each contribute small amounts of risk, and thus additional genetic background influences diagnosis or penetrance of the disease. A similar phenomenon likely underlies changes in SERT Ala56-induced phenotypes when the variant is expressed in mice on different genetic backgrounds (Kerr et al., 2013). The initial studies with SERT Ala56 mice were performed with the Ala56 variant expressed on a 129S6/S4 hybrid background. Unlike the reduced sociability evident for SERT Ala56 in the three-chamber test in these studies, no difference with wildtype littermates was observed in social preference when the variant was expressed on a C57BL/6 background (Kerr et al., 2013). However, social withdrawal in the tube test was observed on either genetic background. As another example, whereas reduced pup vocalizations of SERT Ala56 pups upon maternal separation were observed with the variant on the 129S6/S4 background, *elevated* ultrasonic vocalizations were found for Ala56 pups on a C57BI6/J background. Even further complexity is evident. The maternal genotype of the SERT Ala56 pups appears to impact the penetrance of the SERT Ala56 allele, revealed in biochemical and neurodevelopment comparisons of offspring derived from heterozygous vs. homozygous mothers (Muller et al., 2017). Together, these findings demonstrate a remarkable, context-dependent impact on SERT Ala56 effects, both in terms of direction and penetrance. Such examples are to be expected for a complex disorder like ASD where combinations of genetic changes of small effect drive risk in many subjects.

## Changes in Bowel Function in SERT Ala56 Mice

A common medical comorbidity of ASD is gastrointestinal (GI) dysfunction (McElhanon et al., 2014), with evidence supporting RCTs associated with functional constipation (Marler et al., 2017). The basis for the association of GI symptoms in people with ASD is unclear; however, alterations in the gut-brain axis, the connection of the enteric nervous system (ENS) and CNS may provide some answers (Israelyan and Margolis, 2019). This connection is modulated, in part, through the intrinsic gut 5-HT neuronal system (Gershon, 2013). Additionally, as previously mentioned, 5-HT in the bloodstream is derived from intestinal enterochromaffin cells. Serotonergic neurons of the ENS regulate gut motility as well as the development of the ENS. Changes in gut microbiota can also influence 5-HT availability in the periphery (O’Mahony et al., 2015). SERT Ala56 mice have been found to display diminished colonic transit and electrical wave propagation (Robson et al., 2018), thought to derive from a diminished density of late-born enteric neuronal subsets that can influence gut function and epithelial structure (Margolis et al., 2016). Remarkably, these effects can be reversed by maternal and fetal treatment with the 5-HT_4_ receptor agonist prucalopride (Margolis et al., 2016), consistent with the developmental deficit in synaptic 5-HT signaling in the gut of SERT Ala56 mice, just as predicted for the CNS.

## Genetic and Pharmacological Reversal of Physiological, Behavioral and Gi Alterations in SERT Ala56 Mice

**Figure 4:**
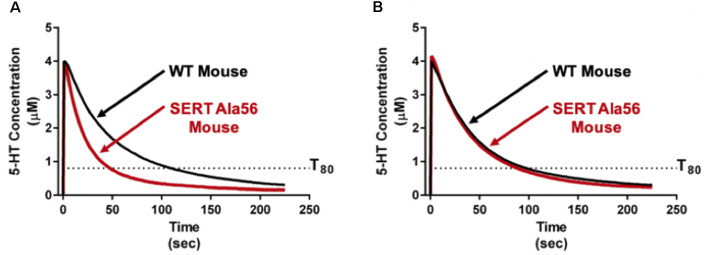
Pharmacological p38α MAPK inhibitor administration normalizes *in vivo* hippocampal 5-HT clearance rates in SERT Ala56 mice. **(A)** SERT Ala56 mice exhibit an elevated rate of hippocampal 5-HT clearance as compared to their SERT Gly56 counterparts when assessed by high-speed *in vivo* chronoamperometry. **(B)** Increased 5-HT clearance rate of SERT Ala56 mice is normalized to WT rates by repeated p38α MAPK inhibition with MW150 (5 mg/kg, QD, i.p. × 7 days). Figure adapted from Robson et al. (2018).

As reviewed above, SERT Ala56 protein *in vitro* and *in vivo* is hyperphosphorylated through a p38 MAPK-dependent pathway. Four genes (α, β, γ, δ) encode isoforms of p38 MAPK. Each of these has a broad tissue distribution and multiple mechanisms of regulation. Previously, we identified that pharmacological blockade of p38 MAPK with SB203580 eliminates the ability of the adenosine A3 receptor to stimulate SERT (Zhu et al., 2004) as well as activation of SERT by the proinflammatory cytokines IL-1β and TNF-α (Zhu et al., 2006). Further work demonstrated that peripheral administration of LPS can rapidly elevate SERT activity *in vivo* through an IL-1 receptor and p38 MAPK-dependent manner (Zhu et al., 2010). Through conditional elimination of p38α MAPK, we demonstrated a requirement for the alpha isoform in serotonergic neurons in LPS-induced SERT stimulation and despair behavior (Baganz et al., 2015), adding evidence for synaptic roles of the kinase (Falcicchia et al., 2020). The implication of the p38α MAPK isoform in SERT regulation encouraged us to consider targeting p38α MAPK to overcome the altered traits of the SERT Ala56 mouse. Fortunately, Watterson’s group had recently generated isoform-selective, CNS-penetrant, p38α MAPK inhibitors (Roy et al., 2019), typified by MW150. Remarkably, after only 1 week of daily i.p. administration of MW150 (Robson et al., 2018), the constitutively elevated hippocampal 5-HT clearance imposed by the SERT Ala56 variant was normalized (Figure 4). Moreover, such treatments normalized multiple pharmacological, behavioral, and GI alterations seen in adult SERT Ala56 mice. Importantly, conditional genetic elimination of serotonergic p38α MAPK in 5-HT neurons, as seen with the rescue of anxiety and despair behavior following LPS treatment, rescued social behavior deficits in the tube test. Together, these findings raise the possibility that some aspects of ASD in some people may be treatable through the use of p38α MAPK inhibitors. The fact that pharmacological inhibition of p38α MAPK can normalize SERT Ala56-induced phenotypes in adult animals suggests also that the circuits modulated by altered 5-HT clearance retain plasticity into adulthood, and indicates that medications based on MW150 may be of benefit to adults on the spectrum.

## Future Directions

Significant evidence now exists to support a physical and functional perturbation of transporter function by the SERT Ala56 mutation, though there remains much to learn about the variant. Understanding the temporal emergence of traits evident in adult SERT Ala56 mice warrants pursuit. Elevated 5-HT clearance induced by the variant has been shown to have the capacity to diminish 5-HT signaling, leading to physiological and behavioral consequences. Given the reversibility of some of these traits with pharmacological manipulations of adult mice, many of these changes appear to influence traits with significant plasticity, including aspects of gut dysfunction that appear to derive from poor, 5-HT dependent development of enteric neural networks. We also do not yet know whether the effects of MW150 are long–lasting after drug discontinuation or can be sustained through chronic administration. Other mouse models of ASD that demonstrate serotonergic dysfunction, particularly those that may feature neuroinflammatory changes, should be explored for utility. Of course, studies in humans to define the safety, tolerability, and efficacy of MW150 are needed and then possibly can be used for individuals with ASD. Our suspicion, given the heterogeneity of ASD, and the highly focused mouse model with which we have tested MW150, is that it will be important to stratify subjects for possible treatment by a relevant biomarker or trait, perhaps hyperserotonemia or RCTs. Gut dysfunction may be a medical comorbidity of ASD that can be treated with agents such as MW150, given our success with the drug in the SERT Ala56 model. As SERT is expressed in both the brain and the gut, it remains possible that certain behavioral traits observed in the SERT Ala56 mice have their origin through primary changes in gut function, possibly through alterations in the microbiome. Animals where the SERT Ala56 mutation is restricted to the brain or periphery are under development and are needed to address this issue. In SERT Ala56 mice, and in the other models discussed above, insufficient attention has been given to the impact of sex as a biological variable. Autism is more common in males than females and evidence indicates that hyperserotonemia is also more common in boys than girls with ASD (Shuffrey et al., 2017), reinforcing our use nearly exclusively of males to date in studies of SERT Ala56 mice. As we have found that peripheral and central 5-HT levels are under the control of different genetic programs in male and female mice (Ye et al., 2014), as has been found in humans (Weiss et al., 2005), we expect that the strength and direction of serotonergic changes with the SERT Ala56 variant or other models of serotonergic dysfunction will be illuminating of potential therapeutic relevance. Last, an extensive network of mRNAs (O’Reilly et al., 2021) and proteins (Quinlan et al., 2020) are altered in the context of SERT Ala56, remarkable given the modest nature of the coding substitution, though reinforcing a functional impact of the variant *in vivo*. Further mining of these networks may provide additional opportunities for insights into disease risk and treatment.

**Figure 5:**
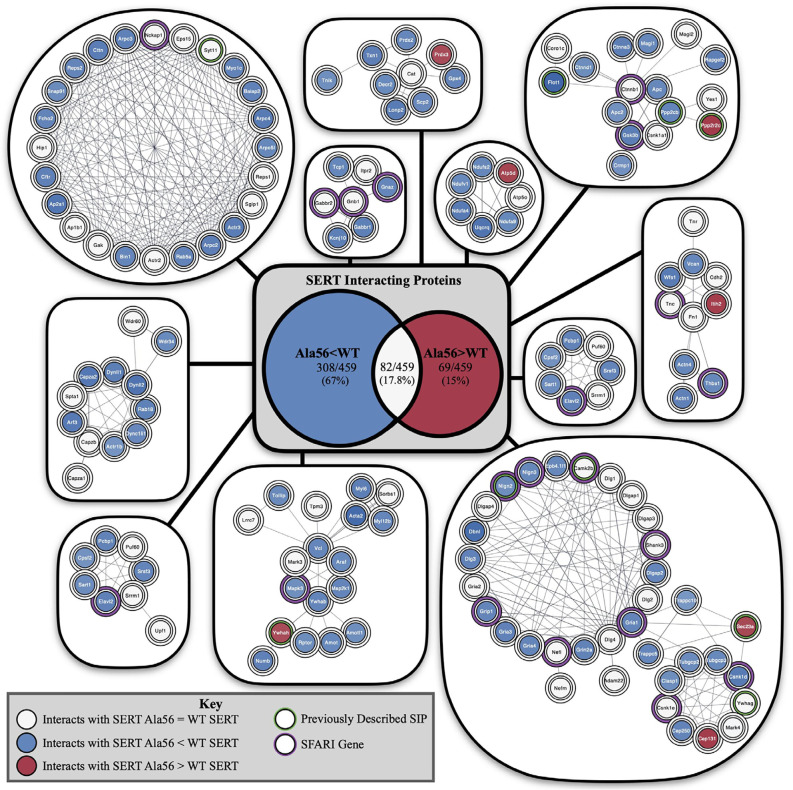
Impact of the Ala56 coding variant on the abundance of SERT-interacting proteins as revealed by a proteomic analysis of midbrain extracts from SERT Ala56 mice. Highlighted proteins previously identified to interact with SERT with a green outline and identified SFARI genes with a purple outline. Center shows Venn diagram of proteins with decreased interactions with SERT Ala56 compared to WT SERT (blue), the number of proteins with increased interactions with SERT Ala56 compared to WT SERT (red) and the number of proteins with similar interaction with both SERT Ala56 and WT SERT (light gray). Figure derived from Quinlan et al. (2020).

Although not studied nearly to the same extent, it is reasonable to expect that other SERT coding variants found to induce SERT hyperfunction *in vitro* would also lead to enhanced SERT-mediated 5-HT clearance and its attendant physiological and behavioral traits when expressed *in vivo*. The degree to which these effects will be modified by genetic background, in the mouse or man, remains to be seen but is likely given the modulatory vs. deterministic nature of 5-HT signaling. By no means does evidence from our studies of rare SERT variants indicate that SERT plays a deterministic role in ASD or even the underlying serotonergic contributions to traits altered in the disorder. However, it is also not unreasonable to consider that multiple sources of ASD risk, genetic or environmental, may impact core or comorbid features of ASD through changes in serotonergic signaling. Support for this idea has recently been provided by studies in the Malenka lab using a 16p11.2 syntenic deletion mouse model of ASD (Walsh et al., 2018). Individuals with this recurrent microdeletion, encompassing approximately 25 genes, were not reported to show distinguishing features that indicate a specific subtype of ASD, though they were noted to more generally display behavioral difficulties involving aggression and overactivity (Kumar et al., 2007). Interestingly, a majority of the genes impacted by the microdeletion were found to associate with a single functional network whose hub gene (TNF), while not residing in the deletion, is an inflammatory cytokine that Zhu et al. (2006) found to positively regulate SERT. In their work, Walsh and colleagues demonstrated deficits in 5-HT secretion in the nucleus accumbens of mice with a 5-HT neuron-specific 16p11.2 deletion to diminished social interactions and that could be rescued by optogenetic stimulation of 5-HT projections to this region, as well as by pharmacological activation of accumbens 5HT_1B_ receptors. Future work with other mouse models of ASD, particularly those demonstrating altered social behavior and/or repetitive behavior should explore opportunities for the rescue of deficits using optogenetic, chemogenetic, or pharmacological manipulation of 5-HT pathways. Recently, we described an altered recovery of SERT-associated proteins in midbrain extracts of SERT Ala56 mice (Quinlan et al., 2020) including a number of ASD linked proteins (Figure 5). These findings suggest that findings with of rare SERT variants may speak to SERT-dependent serotonergic signaling alterations of more common, deterministic ASD genes. Further work to specify SERT protein complexes that participate in serotonergic signaling modulation may uncover novel molecular determinants of risk for ASD and opportunities for therapeutics. A case in point may be our report of ASD-associated A3 adenosine receptor coding variants whose properties include changes in SERT association and an inability to extinguish PKG-dependent SERT regulation (Campbell et al., 2013). Perhaps CNS penetrant, A3 adenosine receptor-specific antagonists might prove of value for some people with ASD. A biomarker and potential therapeutic that is receiving an increasing amount of attention in ASD is oxytocin, and, in humans, Hammock and colleagues have shown levels that negatively correlated with 5-HT. A negative correlation is also seen in oxytocin receptor (*Oxtr)* knockout mice (Hammock et al., 2012).

Our work on SERT coding variants associated with ASD has reinforced our interest in the neurobehavioral impact of PKG and p38α MAPK regulation of SERT in neurobehavioral disorders. In particular, the ability of activation of innate immune signaling pathways to drive SERT hyperfunction and associated behavioral traits *in vivo* suggests new opportunities to target neurobehavioral disorders linked to perturbed 5-HT signaling. Increasingly, we recognize the interplay of immunity and brain development and function, a bidirectional flow that is regulated in both directions by 5-HT (Baganz and Blakely, 2013). Significant evidence indicates that altered innate immunity, favoring a pro-inflammatory state, underlies depression, anxiety, and other mood disturbances, which many models suggest derives from alterations in functional coupling of neural circuits (Raison and Miller, 2011; Felger et al., 2016). We believe that 5-HT and pro-inflammatory signaling are more intertwined than we have understood, made all the more evident by the findings that CNS 5-HT neurons are a significant site of expression in the CNS of receptors for Il-1β (Cunningham et al., 1992; Liu et al., 2019). Early life stress and its induction by limited maternal care may be treatable through consideration of how stress and inflammation impact 5-HT signaling. Maternal immune activation (MIA) can be modeled in rodents and has demonstrated traits in offspring reminiscent of the core symptoms of ASD (Malkova et al., 2012). Whether with respect to developmental disorders or the mood disorders that too often plague us in maturity, we suspect we will continue to learn how genetic and environmental stresses mediate their unwanted effects on the brain and behavior for some time to come.

## Author Contributions

SS and RB wrote the manuscript. All authors contributed to the article and approved the submitted version.

## Conflict of Interest

The authors declare that the research was conducted in the absence of any commercial or financial relationships that could be construed as a potential conflict of interest.
